# Distinct evolutionary lineages of *Schistocephalus* parasites infecting co-occurring sculpin and stickleback fishes in Alaska

**DOI:** 10.1017/S0031182024000593

**Published:** 2024-05

**Authors:** David C. Heins, Kristine N. Moody, Martin C. Arostegui, Brian S. Harmon, Michael J. Blum, Thomas P. Quinn

**Affiliations:** 1 Department of Ecology and Evolutionary Biology, Tulane University, New Orleans, LA 70118, USA; 2 Environmental Sciences Division, Oak Ridge National Laboratory, Oak Ridge, TN 37831, USA; 3 Department of Ecology & Evolutionary Biology, University of Tennessee, Knoxville, TN 37996, USA; 4 Biology Department, Woods Hole Oceanographic Institution, Woods Hole, MA 02543, USA; 5 School of Aquatic and Fishery Sciences, University of Washington, Seattle, WA 98103, USA

**Keywords:** cryptic species, Diphyllobothriidea, molecular phylogeny, parasite, sculpin, stickleback, trophic transmission

## Abstract

Sculpins (coastrange and slimy) and sticklebacks (ninespine and threespine) are widely distributed fishes cohabiting 2 south-central Alaskan lakes (Aleknagik and Iliamna), and all these species are parasitized by cryptic diphyllobothriidean cestodes in the genus *Schistocephalus*. The goal of this investigation was to test for host-specific parasitic relationships between sculpins and sticklebacks based upon morphological traits (segment counts) and sequence variation across the NADH1 gene. A total of 446 plerocercoids was examined. Large, significant differences in mean segment counts were found between cestodes in sculpin (mean = 112; standard deviation [s.d.] = 15) and stickleback (mean = 86; s.d. = 9) hosts within and between lakes. Nucleotide sequence divergence between parasites from sculpin and stickleback hosts was 20.5%, and Bayesian phylogenetic analysis recovered 2 well-supported clades of cestodes reflecting intermediate host family (i.e. sculpin, Cottidae *vs* stickleback, Gasterosteidae)**.** Our findings point to the presence of a distinct lineage of cryptic *Schistocephalus* in sculpins from Aleknagik and Iliamna lakes that warrants further investigation to determine appropriate evolutionary and taxonomic recognition.

## Graphical abstract

**Figure:**
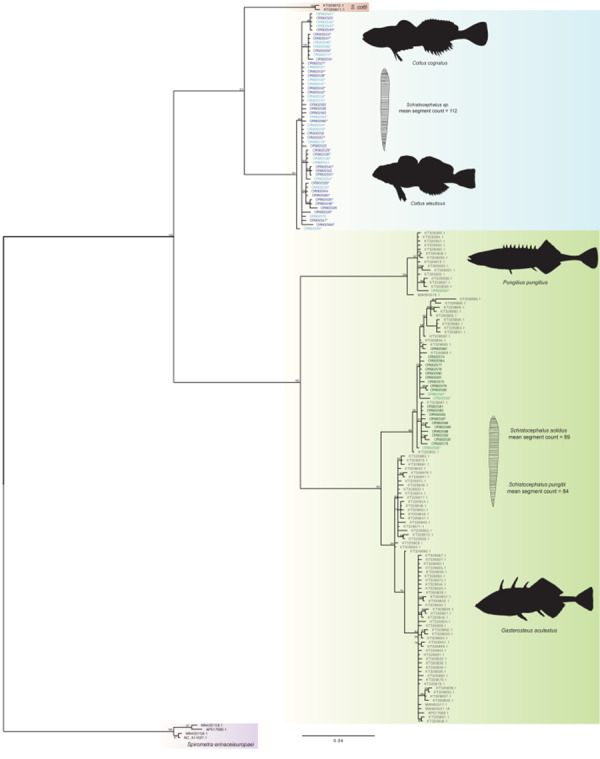

## Introduction

Fish are commonly infected by a diversity of parasites, some of which appear to have subtle or undetectable effects on their hosts (Moles and Heifetz, 1998; Goater *et al*., 2014), whereas others can cause conspicuous host pathology, potentially impacting entire populations and communities (Lafferty, 2008; Heins *et al*., 2010; Goater *et al*., 2014). The selective pressures imposed by parasites on hosts and responses of hosts thereto can result in host specificity, here considered to represent a parasite infecting 1 host species. Although processes of parasitism have received considerable attention in ecological research, the diversity and range of host species remains unclear (Wells and Clark, 2019; Shim *et al*., 2023), especially under conditions allowing for unrestricted transmission of parasites among coincident hosts within a local community (e.g. Blasco-Costa *et al*., 2010; McNamara *et al*., 2014). For example, further investigation might demonstrate that assemblages of sympatric hosts are more frequently infected by phenotypically similar but evolutionarily distinct parasites than is currently known (Choudhury and Scholz, 2020). If so, the diversity of parasites might be underestimated and the structure and function of resident communities mischaracterized.

Research on lineages of tapeworms in the genus *Schistocephalus* (Cestoda: Diphyllobothriidea) may help us to reveal the ecological and evolutionary underpinnings of parasite diversity. These cestode parasites are trophically transmitted with complex life cycles, which is well illustrated by the life cycle of *Schistocephalus solidus* (Smyth, 1962): a free-living, planktonic coracidium larva; followed in turn by a procercoid infecting any of several cyclopoid copepods (first-intermediate host); a plerocercoid infecting a threespine stickleback (*Gasterosteus aculeatus*, second-intermediate host) and an adult worm reproducing in any of about 40 species of piscivorous birds (definitive host). The stickleback fish is the only obligate host in the life cycle. Almost all growth of *S*. *solidus*, from microscopic larva to macroscopic plerocercoid, required for reproduction in the definitive host occurs in the intermediate host fish, which can significantly reduce host energy reserves (Walkey and Meakins, 1970; Lester, 1971; Schultz *et al*., 2006).

Unlike other stages of the *Schistocephalus* life cycle, plerocercoids appear to exhibit strict specificity for particular hosts, notwithstanding ecological conditions one might expect would allow widespread transmission among co-occurring fish species. Notably, research on the first 2 species of *Schistocephalus* demonstrated to be biological species, *S. solidus* and *Schistocephalus pungitii*, indicates that *S. solidu*s infects the threespine stickleback, whereas *S. pungitii* infects the ninespine stickleback (*Pungitius pungitius*) (Nishimura *et al*., 2011). Early morphological and cross-infection studies (Dubinina, 1959; Braten, 1966) provided evidence of host specificity, an inference later supported by phylogenetic analyses showing that distinct lineages of *Schistocephalus* cestodes infect threespine and ninespine stickleback hosts, respectively, from western North America and western Europe (Nishimura *et al*., 2011).

The number of fishes discovered to be intermediate hosts of *Schistocephalus* plerocercoids now includes freshwater sculpins (family Cottidae) from widely separated locations, including bullhead, *Cottus gobio*, in an Arctic river in Finland (Chubb *et al*., 2006); slimy sculpin, *Cottus cognatus*, in lakes of the Arctic region of Alaska, USA (V.B. Holland, unpublished MSc thesis, University of North Carolina at Greensboro, 2006), a lake of southwest Alaska (Harmon *et al*., 2015), Lake Michigan, USA (French and Muzzall, 2008) and the Athabasca River drainage, Alberta, Canada (Braicovich *et al*., 2020) and coastrange sculpin, *Cottus aleuticus*, in a lake of southwest Alaska (Harmon *et al*., 2015). Thus, multiple species of fishes are potentially susceptible to infection by *Schistocephalus* cestodes, including sticklebacks and sculpins that often co-occur in lake habitats (McPhail and Lindsey, 1970). Whether host specificity extends to all or some subset of co-occurring species within local communities of sculpins and sticklebacks is unclear, as is the number of *Schistocephalus* species that may have diversified among fish hosts. Beyond the phylogenetic analyses of Nishimura *et al*. (2011), the only other investigation of host specificity and differentiation in *Schistocephalus* was completed by Chubb *et al*. (2006), who named the cestode *Schistocephalus cotti* as a new species based on morphological and genetic differences between parasites from *C*. *gobio* and *G*. *aculeatus.* One might thus expect *Schistocephalus* plerocercoids of other fish hosts to exhibit morphological and genetic differences indicative of host specificity.

We examined *Schistocephalus* plerocercoids from co-occurring slimy sculpin, coastrange sculpin, threespine stickleback and ninespine stickleback to investigate the nature of host specificity and differentiation among fish hosts in local communities. We drew inferences based on morphological, genetic and phylogenetic comparisons of parasites from all 4 fish species sampled from 2 lakes in different river drainages in southwest Alaska. This effort builds on prior investigations of the ecology (Quinn *et al*., 2012) and genetics (Sprehn *et al*., 2015) of *S. solidus* in threespine stickleback from Bristol Bay (southwest Alaska, USA) that led to detection of cryptic plerocercoids in slimy sculpin and coastrange sculpin from Iliamna Lake (Harmon *et al*., 2015). Initial examinations revealed that the cestodes in the 2 sculpin species exhibit more segments than those in threespine sticklebacks, consistent with the pattern reported for cestodes from cottids in Finland by Chubb *et al*. (2006). Accordingly, we tested the hypothesis that the cestodes infecting sculpin and stickleback hosts correspond to 2 distinct evolutionary lineages. Given prior research illustrating that different species of stickleback hosts carry different species of *Schistocephalus* parasites, we also tested for finer-scale differentiation between sculpin parasites reflecting host specificity sufficient to warrant recognition of distinct species.

## Materials and methods

### Study sites and focal species

Lakes Aleknagik (59.7445 N, 154.1427 W) and Iliamna (59.3435 N, 154.7802 W) are part of the Wood River and Kvichak River watersheds, respectively, both of which drain into Bristol Bay, Alaska. Lake Aleknagik is smaller (83 km^2^ in surface area, 3.6 km^3^ in volume, with mean and maximum depths of 43 and 110 m) than Iliamna Lake (2622 km^2^ in area, 115.3 km^3^ in volume, with mean and maximum depths of 44 and 301 m; Burgner *et al*., 1969). Both lakes are oligotrophic but primary and secondary production levels are higher in Aleknagik than Iliamna (Burgner *et al*., 1969). The zooplankton communities are similar (primarily cyclopoid and calanoid copepods and cladocerans) but Aleknagik has a higher proportion of *Daphnia* than does Iliamna, where *Bosmina* is the dominant cladoceran (Hoag, 1972; Carter and Schindler, 2012; T. P. Quinn, unpublished data, 2024). In boreal freshwater ecosystems, ninespine and threespine sticklebacks and slimy and coastrange sculpins frequently co-occur (McPhail and Lindsey, 1970).

### Sample collection

Threespine and ninespine stickleback were sampled from multiple locations in the limnetic and littoral zones whereas coastrange and slimy sculpins were sampled from littoral zone sites in both lakes in August and September of 2012–2015 and 2017–2019. Limnetic sampling was conducted with a towed surface net at a series of long-term monitoring sites in open water (see Arostegui *et al*., 2018 for details). Littoral sampling was conducted with a hand net, beach seine or baited traps along mainland or island shorelines. Specimens were euthanized after capture with an overdose of buffered MS-222 and dissected for removal and evaluation of all *Schistocephalus* parasites, which were found in the body cavities. Sculpin species were identified with a dissecting microscope by the number of chin pores present: 1 – coastrange, 2 – slimy (Morrow, 1980). Due to wide variation in size among parasites found in fish hosts, segments were only counted (under a dissecting microscope) for specimens large enough to permit an accurate count. Parasite specimens and fish hosts were preserved in 70% ethanol and stored at room temperature.

### Meristic analysis

To determine whether there was meristic evidence of parasite host specificity and differentiation (Chubb *et al*., 2006), parasite segment counts were compared according to host fish species using a generalized least squares (GLS) regression model to account for unequal sample sizes of *Schistocephalus* parasites from slimy sculpin, threespine stickleback and ninespine stickleback in both lakes, and from coastrange sculpin in Iliamna Lake (Table 1). The absence of *Schistocephalus* parasites in coastrange sculpin sampled from Aleknagik Lake also precluded formal testing for a host–lake interaction effect on segment counts in the model. Thus, a combined factor of host/lake (e.g. Iliamna slimy sculpin, Aleknagik slimy sculpin) was tested to account for potential between-lake variation within host species when comparing segment counts among host species. To identify the best-fit GLS model, variance structures were first compared for host, lake and host/lake in models with host/lake as a main effect. Backward selection was then conducted on the main effect following Zuur *et al*. (2009). Model selection (including identification of the optimal variance structure) was conducted with Akaike's information criterion (AIC – Akaike, 1974) of maximum-likelihood estimates. The identified best-fit model was then re-estimated with restricted maximum likelihood. Pairwise comparisons among host/lake combinations were conducted with Tukey multiple comparison tests using a Benjamini–Hochberg correction. Models were built and validated in R version 3.6.3 using the following packages: ‘stats’ (R Core Team, 2020), ‘nlme’ (Pinheiro *et al*., 2016), ‘piecewiseSEM’ (Lefcheck *et al*., 2018) and ‘multcomp’ (Hothorn *et al*., 2008).

**Table 1.** Summary metrics of *Schistocephalus* parasite segment counts in different fish species from Iliamna Lake and Lake Aleknagik, Alaska

Host species	Lake	Sample size	Mean (s.d.)	Range
Coastrange sculpin	Iliamna	104	118 (18)	36–154
	Aleknagik	–	–	–
	Combined	–	–	–
Slimy sculpin	Iliamna	24	114 (16)	83–142
	Aleknagik	12	106 (11)	83–123
	Combined	36	111 (15)	83–142
Threespine stickleback	Iliamna	91	84 (11)	60–107
	Aleknagik	6	97 (10)	83–112
	Combined	97	85 (11)	60–112
Ninespine stickleback	Iliamna	21	82 (8)	69–103
	Aleknagik	17	85 (8)	65–100
	Combined	38	84 (8)	65–103

Sample size is the number of parasites examined; mean (s.d.) and range refer to the number of segments per parasite.

### Genetic sequencing and analysis

To quantify genetic variation and potential differentiation of *Schistocephalus* plerocercoids across host species, genomic DNA was first extracted from 77 parasite specimens (20 from slimy sculpin, 33 from coastrange sculpin, 20 from threespine stickleback and 4 from ninespine stickleback), using the Qiagen DNeasy Blood and Tissue Kit according to the user manual for tissue extraction. DNA concentrations were quantified using a Nanodrop Spectrophotometer and then standardized to 20 ng μl^−1^. Polymerase chain reactions (PCRs) using GoTaq polymerase were performed to amplify a ~1100 bp portion of the NADH1 mitochondrial gene using primers from Nishimura *et al*. (2011) (forward: NAD 9F1 – GGGTTTGCGTCTCGGAGATGGTG; reverse: NAD 3R1 – GCGTAATCGTTGGTGGAAC). PCR amplifications involved an initial cycle of denaturation of 94°C for 3 min, 35 subsequent cycles of denaturation at 94°C for 1 min, annealing at an optimized temperature of 56°C for 1 min and extension at 72°C for 1 min, followed by a final extension step of 72°C for 10 min. Post-PCR products were cleaned using ExoSap (Thermo Fisher Scientific, Waltham, MA, USA). The resulting cleaned-PCR products were cycle-sequenced with each primer used for PCR amplification. Sanger electrophoresis was conducted on an ABI 3100xl. Sequences were cleaned and trimmed using Sequencher v5.1 (Gene Codes Corporation, Ann Arbor, MI, USA). All subsequent analyses focused on a 396 bp section that excluded low-quality and non-overlapping forward and reverse sequences of the target region. The haplotype of each parasite specimen was then determined according to nucleotide sequence variation. Nucleotide sequences representative of each unique haplotype were subsequently deposited in the GenBank database (accession numbers OR902521–OR902597).

Estimates of genetic variation and differentiation were determined according to nucleotide sequence variation. First, haplotype sequences were run through NCBI Blastn (Altschul *et al*., 1990) to scan for homologous nucleotide sequences. Haplotype sequences also were run through Blastx for translated amino acid homology. Sequence divergence, haplotype diversity (*h*), number of segregating sites (*S*) and nucleotide diversity (*π*) were estimated in DnaSP 6.12.03 (Rozas *et al*., 2017). Phylogenetic analyses were conducted on an alignment of the newly generated sequences and GenBank repository sequences of the NADH subunit ND1 gene from *S. solidus*, *S. pungitii*, *S. cotti* and *Spirometra erinaceieuropaei* (outgroup). All sequences were aligned with Clustal Omega (Goujon *et al*., 2010) as implemented in Sequencher v. 5.1. Bayesian analysis of the alignment was performed with MrBayes 3.2.7a (Ronquist *et al*., 2012) using a general time-reversible model with a portion of invariable sites and gamma-shaped distribution of rates across site models (GTR + I + Γ) and 2 simultaneous Markov chain Monte Carlo analyses with 4 chains for 3 × 10^6^ generations. Trees were sampled for every 1000 generations, with a 25% burn-in and stop rule once convergence was established with the final deviation of split frequencies fell below 0.01.

## Results

### Meristic comparison

**Figure 1.**
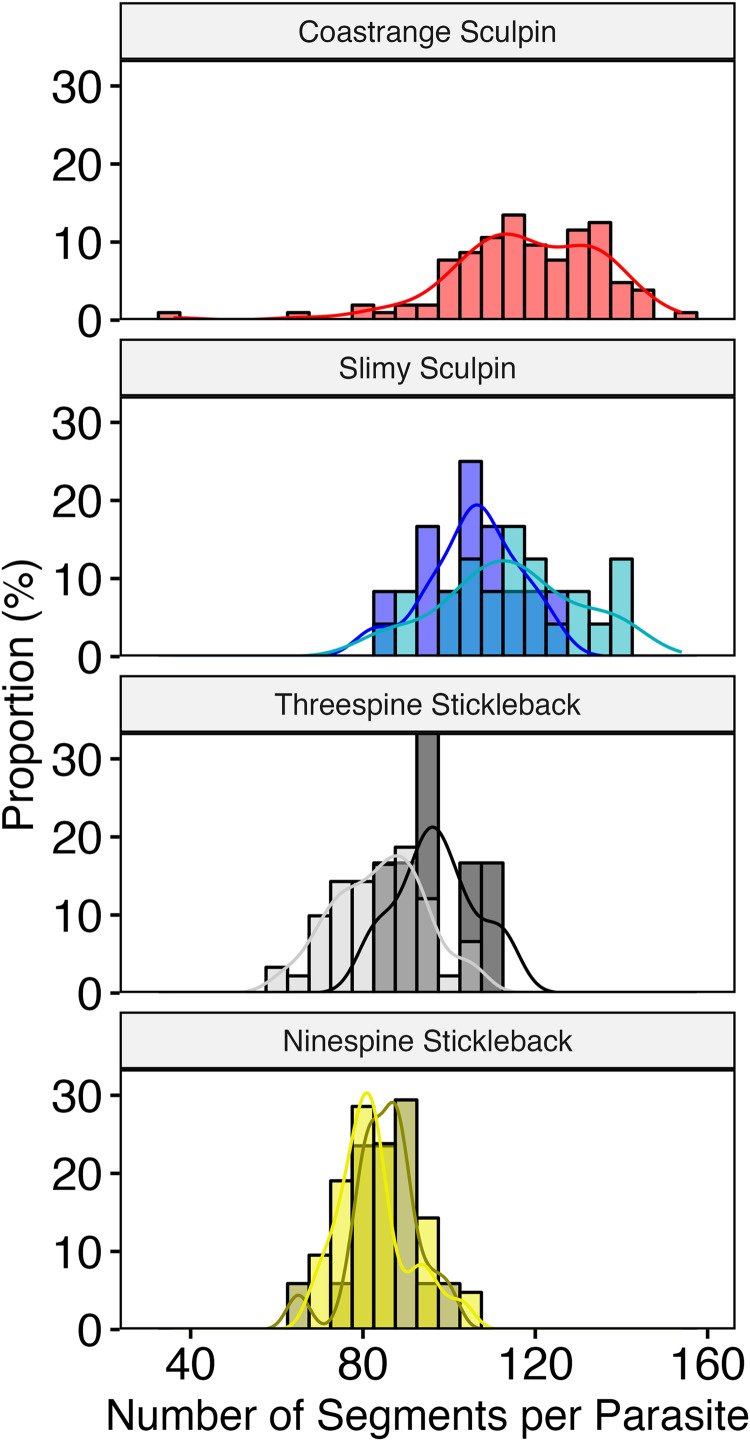
Number of segments per *Schistocephalus* parasite by host fish species. Within panels, the lake-specific data are presented as colour-coded, overlapping distributions (lighter shade – Iliamna; darker shade – Aleknagik; intermediate shade – overlap) with their corresponding probability density functions. Coastrange sculpins with parasites were only collected at Iliamna Lake.

Overall, *Schistocephalus* parasites from the 2 stickleback species (*n* = 135) had fewer segments than the parasites in the 2 sculpin species (*n* = 140) (Table 1); 92.6% of the cestodes in sticklebacks had <100 segments and 85% of those in sculpins had >100 segments (Fig. 1). Regression analysis of parasite segment counts indicated a main effect of host/lake (*F* = 60.6, *P* < 0.0001) and a variance structure for host in the GLS model with the lowest AIC score and highest AIC weight (Table 2). The best-fit model (pseudo-*R*^2^ = 0.56) identified large, significant differences in the mean segment counts between the 2 stickleback species and the 2 sculpin species both between and within lakes, except between slimy sculpin and threespine stickleback in Aleknagik Lake (Table 3). There were smaller but significant differences between lakes in segment counts of parasites from each stickleback species (e.g. threespine stickleback from Iliamna and Aleknagik), and between the stickleback species (threespine and ninespine), both within and between lakes (Table 3). In contrast, small, but significant, differences in parasite segment counts between coastrange and slimy sculpin only occurred between lakes (Table 3). That is, segment counts did not significantly differ between the cestodes in the 2 sculpin species within the lake (Iliamna) where such a comparison was possible (the absence of cestodes in coastrange sculpin sampled from Aleknagik Lake precluded comparison to those in slimy sculpin within that lake).

**Table 2.** GLS model selection results for parasite segment counts, including the difference in AIC relative to the model with the lowest score (ΔAIC) and the AIC weight (AICw)

Main effect	Variance structure	ΔAIC	AICw
Host/lake	Host	0.0	0.84
Host/lake	Host/lake	3.3	0.16
Host/lake	Lake	30.9	0.00
Host/lake	None	40.7	0.00
Host/lake	Host	0.0	1.00
Null	Host	198.6	0.00

Rows above the dashed line describe the optimal variance structure, whereas rows below describe the subsequent main effect selection in models with the optimal variance structure.

**Table 3.** Pairwise, model-predicted differences in segment counts among host/lake combinations

	A – Sl Sc	A – 3-sp	A – 9-sp	I – Cr Sc	I – Sl Sc	I – 3-sp
A – 3-sp	−9 (−21 to 3)	–				
A – 9-sp	−21 (−30 to −11)***	−12 (−21 to −2)*	–			
I – Cr Sc	12 (3–21)*	21 (11–30)***	32 (27–38)***	–		
I – Sl Sc	8 (−2 to 18)	17 (7–27)**	29 (22–36)***	−3 (−10 to 3)	–	
I – 3-sp	−22 (−30 to −13)***	−13 (−22 to −4)**	−1 (−6 to 3)	−34 (−38 to −29)***	−30 (−36 to −24)***	–
I – 9-sp	−23 (−32 to −14)***	−15 (−24 to −5)**	−3 (−8 to 3)	−35 (−40 to −30)***	−32 (−38 to −25)***	−1 (−6 to 3)

Host: 3-sp, threespine stickleback; 9-sp, ninespine stickleback; Cr Sc, coastrange sculpin; Sl Sc, slimy sculpin. Lake: A, Lake Aleknagik; I, Iliamna Lake.

The mean difference (95% confidence interval) of each comparison is rounded to the nearest integer, and is calculated as the difference between the corresponding host/lake of that row minus the host/lake of that column (e.g. Aleknagik threespine stickleback, on average, exhibit 9 less segments than Aleknagik slimy sculpin). The comparison type is colour coded: within a species and between lakes – yellow, among species and within a lake – grey, among species and lakes – white.

Comparison *P* value: <0.05 (*), <0.01 (**), <0.001 (***).

### Genetic variation and phylogenetic divergence

Parasites from sculpin hosts (accession numbers OR902521–OR902573) had 23 haplotypes with a haplotype diversity of 0.94, 28 segregating sites and a nucleotide diversity of 0.007. NCBI Blast analysis recovered 89.25% sequence similarity to *S. cotti* (accession numbers KT326912.1 and KT326911.1). Eighteen haplotypes among the parasites from stickleback hosts exhibited a haplotype diversity of 0.96, 60 segregating sites and a nucleotide diversity of 0.02. NCBI Blast analysis recovered a 95% similarity between parasites from threespine sticklebacks (accession numbers OR902574–OR902593) to *S. solidus* (accession numbers MW602517.1, MW602521.1 and AP017669.1) and there was a 98.74% similarity between 1 parasite from a ninespine stickleback (accession number OR902594) to *S. pungitii* (accession number MW602516.1), whereas the other 3 parasites from ninespine sticklebacks (accession numbers OR902595–OR902597) had only a 86.48% similarity with *S. pungitii* (accession number MW602516.1), but a 94% similarity with *S. solidus* (accession numbers MW602517.1, MW602521.1 and AP017669.1). Nucleotide sequences from coastrange and slimy sculpin parasites were similar (overall sequence divergence of 0.7%) whereas there was 4% sequence divergence between parasites from threespine and ninespine stickleback hosts. Notably, there was 20.5% nucleotide sequence divergence between parasites from sculpin and stickleback hosts. Amino acid similarity was 90% between the parasites from sculpin hosts and *S. cotti*, 85% between the parasites from sculpin hosts and *S*. *solidus* (accession numbers QXU59603.1 and QXU59651.1), and there was a 86% similarity between parasites from sculpin hosts to *S. pungitii* (accession number QXU59591.1).

**Figure 2.**
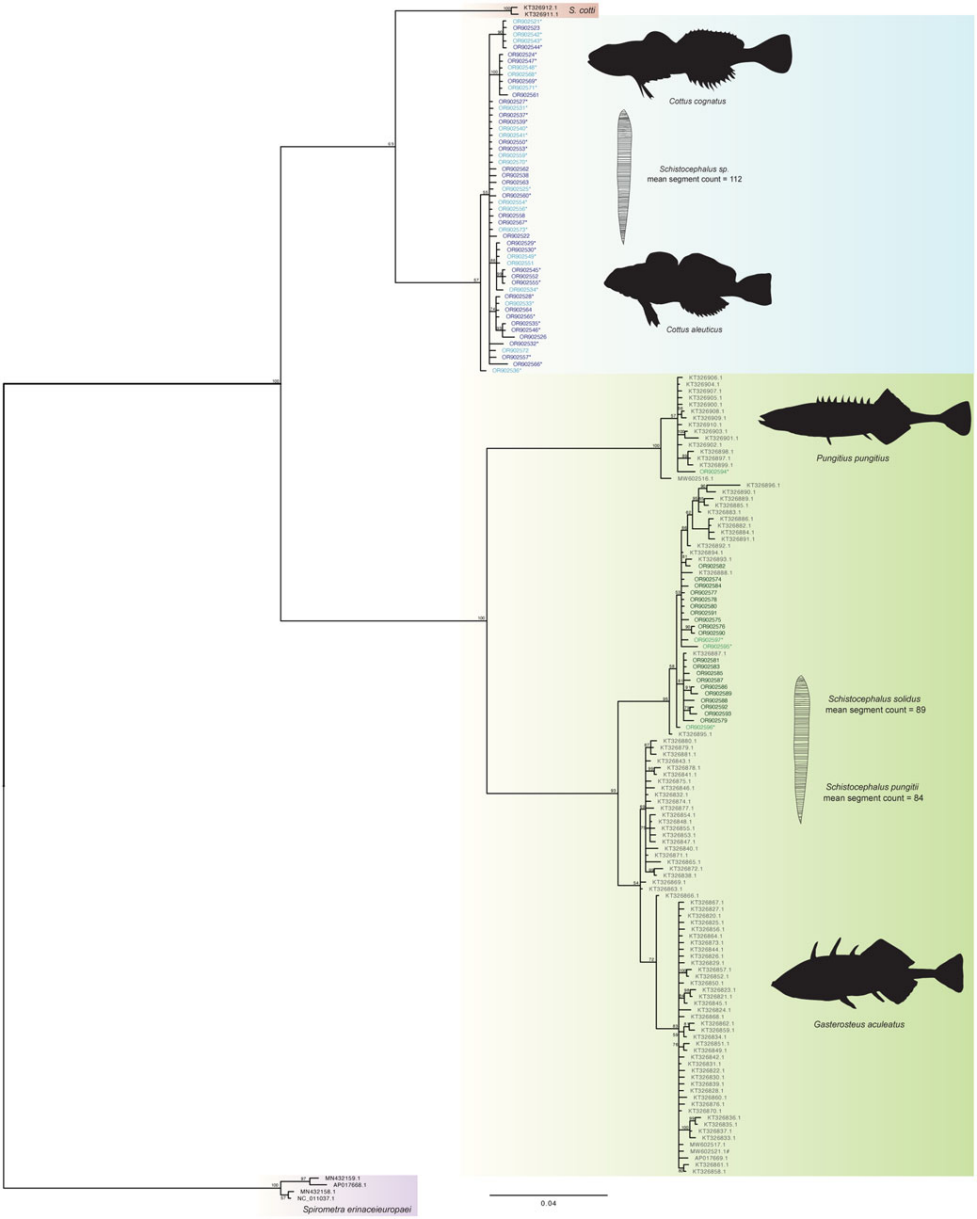
Bayesian tree (scale bar: 0.02 estimated substitutions per site) of *Schistocephalus* parasites sequenced with partial NADH1 gene from their respective host fish species: *Cottus cognatus* parasites (*n* = 20), light blue; *Cottus aleuticus parasites* (*n* = 33), dark blue; *Gasterosteus aculeatus parasites* (*n* = 20), dark green and *Pungitius pungitius* parasites (*n* = 4), light green. * denotes corresponding segment counts were obtained from the individual; ^#^ denotes *Pusa hispida botnica* host. Fishes and parasites are not drawn to scale.

Bayesian phylogenetic analysis recovered 2 distinct clades (Fig. 2), one composed of parasites found in sculpin hosts and the other of parasites found in stickleback hosts. The 2 clades were separated by approximately 20% sequence variation without ambiguity. Neither lake nor collection year moderated the tree structure – all sculpin derived parasites clustered within the sculpin clade and likewise, all stickleback derived parasites clustered together. Support was not found for distinct clusters of parasites from threespine and ninespine stickleback hosts, respectively, nor for parasites clustering according to sculpin host species (Fig. 2).

## Discussion

Here we provide evidence of host specificity and differentiation among *Schistocephalus* plerocercoids infecting a complement of co-occurring host species. All 4 fish hosts are either regularly or incidentally susceptible to infection *via* trophic transmission within the local community. Infections conceivably could have arisen from non-specific transmission whereby all hosts were infected by the same parasite. To the contrary, our results indicate that infection is moderated by host specificity, where evolutionarily distinct *Schistocephalus* parasites infect different intermediate host species. *Schistocephalus* from sculpins differed from those in sticklebacks, supporting prior research pointing to host specificity. Chubb *et al*. (2006) proposed that *Schistocephalus* infecting cottids are evolutionarily distinct from those in threespine sticklebacks based on significant differences in mean segment number and PCR amplification trials suggestive of nucleotide sequence divergence. We detected similar meristic differences, and our genetic and phylogenetic analyses revealed that *Schistocephalus* plerocercoids from cottids are highly differentiated from those in sticklebacks, bolstering the argument for recognizing *Schistocephalus* infecting cottids as 1 or more distinct evolutionary lineages (i.e. species). We did not recover clear evidence of finer-scale evolutionary divergence, but our findings are nonetheless broadly consistent with phylogenetic evidence that *Schistocephalus* diversification corresponds to host species specificity (Nishimura *et al*., 2011). A phylogeny based on mtDNA sequence variation recovered distinct clades of *Schistocephalus* infecting threespine stickleback and ninespine stickleback, supporting the hypothesis (Dubinina, 1959) that *S*. *solidus* and *S*. *pungitii* represent 2 distinct evolutionary lineages warranting species recognition. Nishimura *et al*. (2011) also found differences despite the potential for substantial gene flow among parasites in areas supporting populations of both sticklebacks, suggesting that *S*. *solidus* and *S*. *pungitii* are good biological species. Mounting additional efforts to build on our study would likely shed further light on the specificity of *Schistocephalus* parasites within and among intermediate fish hosts.

The morphological phenotypes of plerocercoids from the 2 stickleback species were distinct from the plerocercoids infecting the 2 sculpin species. There were significant differences in mean segment count for all comparisons within and between lakes, except for the low sample size comparison between slimy sculpin (*n* = 12) and threespine stickleback (*n* = 6) in Aleknagik Lake. Though compelling, a difference in segment counts is neither indisputable evidence of evolutionary differentiation nor can it serve as a definitive basis for taxonomic identification. Prior research has questioned the importance and use of segment counts as a diagnostic attribute. Both Clarke (1954) and Dubinina (1980) concluded that segment number of fully segmented young plerocercoids exhibits little increase with further growth, and Dubinina (1980) suggested that segment number is a genetically determined trait. Chubb *et al*. (1995), however, concluded that segment number is phenotypically plastic and related to plerocercoid size. Chubb *et al*. (2006) later proposed that plerocercoid and adult segment number could be used to identify *Schistocephalus* species and included the trait in their taxonomic key to plerocercoids of *Schistocephalus* species. Further study of this trait is warranted; experimental research (e.g. a common garden experiment) to evaluate heritability and plasticity could be especially informative.

Phylogenetic analysis recovered 2 well-supported monophyletic clades, with approximately 20% nucleotide sequence divergence separating *Schistocephalus* infecting sticklebacks from those in sculpin hosts. Membership in the clades did not vary according to sampling location or year. The estimated percentage of divergence is widely associated with species- or higher levels of taxonomic differentiation. For example, there is only 1.24% genome-wide sequence divergence between humans and chimpanzees (Ebersberger *et al*., 2002), and ~2% mtDNA sequence divergence is widely used for affirming or recognizing species of freshwater fish (Blum *et al*., 2008). We detected no ambiguous sequences between stickleback- and sculpin-derived parasite clades (no detection of any sculpin parasites in sticklebacks nor any stickleback parasites in sculpins), indicating that differentiation is not recent and that hybridization has likely not occurred between members of these 2 clades. Notably, the observed sequence variation translated to 18–20 amino acid differences between our sequenced sculpin host parasites and GenBank-derived stickleback host parasites (both 3-spine and 9-spine hosts), which offers further support for recognizing the sculpin and stickleback parasite groups as distinct evolutionary lineages. In comparison, Nishimura *et al*. (2011) proposed recognizing 2 different parasite species in threespine and ninespine sticklebacks (respectively) based on lower levels of sequence divergence. Although our phylogenetic analysis demonstrates reciprocal monophyly between parasites from *P. pungitius* and *G. aculeatus* from GenBank sequences, we did not detect a clear pattern of divergence among our parasites of the 2 stickleback species. All of our *G. aculeatus* sequences grouped within the *S. solidus* clade; however, for *P. pungitius*, only one of our sequences, out of four, grouped within the *S. pungitii* clade. This may be an artefact of analysing a relatively short region of the NADH1 gene that provided less information on sequence variation than the region examined by Nishimura *et al*. (2011). Empirical investigations to date support the conclusion that *S*. *solidus* and *S*. *pungitii* are only able to infect their respective, specific host species of stickleback (Nishimura *et al*., 2011; Henrich *et al*., 2013). Nonetheless, the ability to hybridize the 2 species of *Schistocephalus in vivo* suggests that hybridization in nature within a single host may be possible (Henrich *et al*., 2013). We also did not detect a clear distinction between slimy and coastrange sculpin parasites, but we cannot exclude the possibility that the parasites comprise distinct evolutionary lineages among sculpin host species.

Further investigation focusing on these questions and on diversity among *Schistocephalus* parasites is warranted, particularly among parasites from sculpin hosts. Attention should also be given to *Schistocephalus nemachili* and *Schistocephalus thomasi*, which are considered valid species (Global Cestode Database), although not well studied. Our efforts were constrained in part by the utility of primers for PCR amplification and conventional Sanger sequencing. Published primer sets that work well for stickleback parasites do not perform as well for sculpin parasites. Chubb *et al*. (2006) encountered similar challenges with microsatellite primers designed for *Schistocephalus* from threespine stickleback that did not amplify for parasites infecting bullhead, *C*. *gobio*. Accordingly, further investments should be made to develop primers and molecular markers for parasites derived from different host species. This would allow for broader sequencing of the full NADH1 gene with (putatively) lineage-specific primers. Next-generation sequencing (e.g. ddRAD single-nucleotide polymorphism analysis) could also provide greater resolution to clarify species- or population-level differences, as well as finer-scale patterns of host specificity, host–parasite evolution and trophic transmission in *Schistocephalus*.

Further investigation could lead to *Schistocephalus* being recognized and adopted as a system for studying speciation in parasites. Parasites in the diphyllobothriidean cestode genus *Ligula* have been the subject of more and more comprehensive investigations of evolutionary differentiation among parasites. Research thus far has revealed evidence of diversification corresponding to fish hosts and geography. Nazarizadeh *et al*. (2023), for example, found strong support for 10 or more evolutionary lineages reflecting taxonomic distinctions (i.e. genera and orders) of fish hosts, including groups that differ in global extent. Differences in geographic distributions offer opportunities to study vicariant and ecological speciation among parasites (Nazarizadeh *et al*., 2023). As shown in previous studies (Sprehn *et al*., 2015; Strobel *et al*., 2016), *S. solidus* does vary genetically across different geographic regions and could explain the phylogenetic patterns within our *S. solidus* clade (Fig. 2). Unfortunately, geographic data are not available for the sequences obtained through GenBank that start with KT. Additional geo-referenced sampling and sequencing could help clarify these patterns, the potential drivers of genetic variation and potentially cryptic divergence. Discovering cryptic species is important to gaining greater insight into community structure and function, as well as processes of evolutionary biology and biogeography (Pérez-Ponce de León and Nadler, 2010; Nadler and Pérez-Ponce de León, 2011). Revealing crypsis through modern molecular methods is especially important for parasites that are morphologically simple with few diagnostic characteristics (Hanelt *et al*., 2015), and it is even more so for morphologically simple parasites with unreliable morphological traits such as *Schistocephalus*. Our findings illustrate that research on *Schistocephalus* parasites is a potentially fertile area of enquiry using state-of-the-art molecular tools to manifest findings that complement those from ongoing research on *Ligula*.

In addition to the opportunities for further research on the parasites themselves, our study highlights the need for more information on the possible mode of infection of sculpins by *Schistocephalus* parasites. Sampling of coastrange and slimy sculpin from Iliamna Lake has not revealed any zooplankton in the diets ( P. B. Roger, unpublished MSc thesis, University of Washington, 1971; B. S. Harmon, unpublished data, 2012). A literature review of coastrange and slimy sculpin food studies from other North American lakes either did not uncover zooplankton in the diet or found it to be a very minor component. Only 1 study mentioned cyclopoid copepods (Bunnell *et al*., 2015). Consumption of cyclopoid copepods, the intermediate host of *Schistocephalus*, appears to be very limited among fish 20–100 mm standard length, the size range primarily sampled in the aforementioned studies. Other freshwater sculpin species in lakes elsewhere substantially consume cyclopoid copepods but apparently only seasonally as young-of-the-year (YOY) fish <20 mm standard length (Broadway and Moyle, 1978; D. Neverman, Unpublished MS Thesis, Utah State University, 1989). Similarly, threespine stickleback become infected seasonally soon after hatching as YOY (Heins *et al*., 2016; Wohlleben *et al*., 2022). We hypothesize that coastrange and slimy sculpins also become infected seasonally soon after birth as YOY fish. Further research on the trophic ecology of sculpins, especially their consumption of zooplankton and means of infection, remains a critical area of investigation. Systematic investigations of the trophic ecology, linked to infection rates, for both sculpin species in a range of habitats would be fruitful. They occur in streams and lakes, for example, but the extent of movement between these habitats is unclear. In addition, better information on the comparative ecology (diet and habitat use patterns) of the 2 stickleback species, and the key avian predators for all these species would be informative.

In conclusion, an integrative systematic approach combining ecological, morphological and genetic data supports the hypothesis that parasites infecting coastrange and slimy sculpins in Aleknagik and Iliamna lakes of Alaska are biologically distinct, apart from the 2 known species of parasites infecting ninespine and threespine sticklebacks. Our goal was to test for these differences and to summarize what is known about the evolutionary diversification of cestodes in the genus *Schistocephalus*. These parasites offer a challenging and potentially enlightening investigation into adaptive radiation. For example, we do not know whether the parasites in coastrange and slimy sculpins we studied represent 2 separate species, nor whether any of those parasites differ from *S. cotti*. The species-level host specificity thus far observed for parasites infecting sticklebacks suggests that there may be 3 biological species infecting the sculpins known to be parasitized by *Schistocephalus*. The results of this investigation should inform future research and provide a foundation for detailed systematic studies of diversity and dynamics of the evolutionary pattern presented by the genus *Schistocephalus*.

## Data Availability

Sequence data are available in GenBank (accession numbers OR902521–OR902597) (upon publication).
